# Association of multiple anthropometric indices with in 944,760 elderly Chinese people

**DOI:** 10.4178/epih.e2023046

**Published:** 2023-04-17

**Authors:** Lirong Dong, Yuanyuan Wang, Jinshui Xu, Yang Zhou, Guiju Sun, Dakang Ji, Haijian Guo, Baoli Zhu

**Affiliations:** 1 Department of Integrated Services, Jiangsu Provincial Center for Disease Control and Prevention, Nanjing, China; 2 Key Laboratory of Environmental Medicine and Engineering of Ministry of Education, School of Public Health, Southeast University, Nanjing, China; 3 Center for Global Health, School of Public Health, Nanjing Medical University, Nanjing, China

**Keywords:** Hypertension, Anthropometric indices, Aging

## Abstract

**OBJECTIVES:**

The aims of this study were to update the latest data on the prevalence of hypertension (HTN) in the elderly Chinese population and to assess relationships between new anthropometric indices and HTN.

**METHODS:**

Data were obtained from the Basic Public Health Service (BPHS) survey for Jiangsu Province, China. A total of 944,760 people aged 65 years and older were included in this study. Blood pressure was measured by trained investigators. Body weight, body mass index (BMI), waist circumference (WC), waist-to-height ratio (WtHR), conicity index (COI), body roundness index (BRI), and a body shape index (ABSI) were included in the analysis as anthropometric indices. Logistic regression analysis and restricted cubic splines were used to evaluate the association of anthropometric indices with HTN.

**RESULTS:**

The prevalence of HTN among elderly residents of Jiangsu Province was 64.7% (95% confidence interval, 64.6 to 64.8). After adjusting for multiple covariates, all anthropometric indices except ABSI showed significant non-linear positive dose-response associations with HTN across sex (p_nonlinear_<0.001). Among participants with BMI <28 kg/m^2^, abnormal weight, WC, WtHR, BRI, COI, and ABSI were positively associated with HTN.

**CONCLUSIONS:**

The prevalence of HTN in the elderly in Jiangsu Province is gradually increasing. It is necessary to consider the combination of ABSI and COI with BMI for screening elderly individuals for HTN in follow-up prospective studies.

## INTRODUCTION

Hypertension (HTN) is a major risk factor for failure of the heart, brain, kidney, and other vital organs [1,2]. The 2019 Global Burden of Disease Study shows that high systolic blood pressure (SBP) is the most prominent factor contributing to total mortality in male and female worldwide among all level 2 risk factors [3]. HTN is widely prevalent worldwide. More than 1 male in 4 males and 1 female in 5 females suffer from HTN, and its prevalence is higher in the elderly [4,5].

In 2011, World Health Organization signed the Global Action Plan for the Prevention and Control of Non-communicable Diseases, which aimed to reduce the prevalence of HTN by 25% between 2010 and 2025 [6]. Committed to achieving this goal, China has made the reduction of HTN prevalence an important part of its public health policy.

Obesity, commonly defined internationally by body mass index (BMI), is considered one of the most important risk factors for HTN [7,8]. Although the mechanism of how obesity causes elevated blood pressure (BP) is unclear, obesity and HTN have been linked epidemiologically [9,10]. This association also exists in low-income countries [11]. However, some researchers have argued that considering BMI as the only accurate predictor of chronic disease risk is flawed, since it does not reflect body fat distribution [12,13]. Some indices that reflect central obesity, such as the waist-to-height ratio (WtHR), a body shape index (ABSI), and waist circumference (WC), also seem able to predict the likelihood of HTN [13,14]. Nevertheless, the predictive power of these measures needs to be investigated further. Additionally, the degrees of association between different anthropometric indices and HTN risk and the shapes of potential non-linear dose-response relationships remain uncertain.

According to China’s Seventh National Population Census, the demographic group aged 65 years and older has reached 190.59 million, accounting for 13.5% of the total population, and the dual pressures of aging and chronic diseases continue to increase the overall economic burden on society [15]. For Jiangsu Province, which has one of China’s highest proportions of aging individuals, the imbalanced allocation of medical resources is very likely to result in a waste of medical resources and effective medical care for patients with HTN. Therefore, this study intended to take Jiangsu Province as an entry point to examine the distribution of HTN in different geographical groups of elderly residents and to clarify the shapes of the potentially non-linear dose-response relationships between different anthropometric indices and HTN, which are essential for the development of more detailed guidelines for the primary prevention of HTN.

## MATERIALS AND METHODS

### Study design and participants

**Figure 1.**
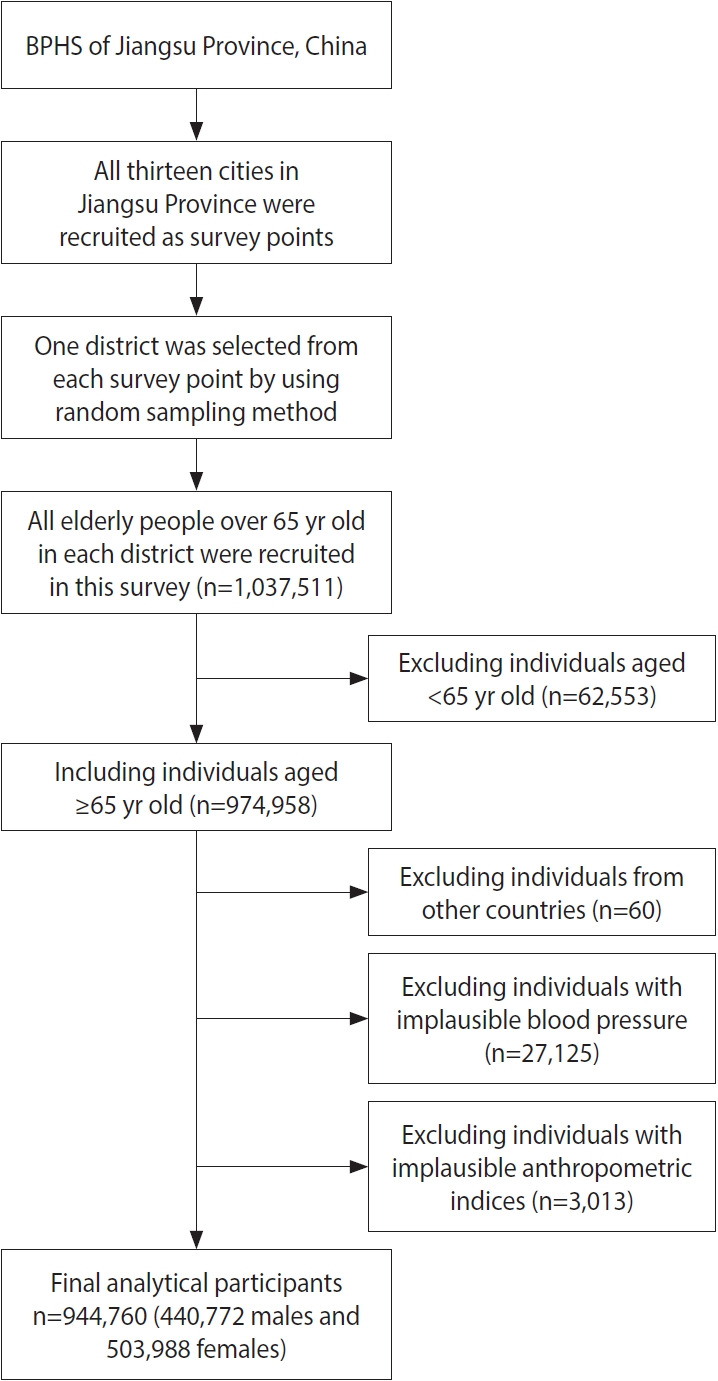
Flowchart of the research participants from Basic Public Health Service (BPHS) in Jiangsu Province, China.

The landmark health policy Basic Public Health Services (BPHS), subsidized by national and local government budgets, has been in place since 2009 [16,17]. BPHS provides free primary healthcare services, including lifestyle and health status assessments, physical examinations, and health guidance, to residents who have lived in the local jurisdiction for at least 6 months. According to BPHS, primary-care facilities are required to screen residents aged 65 years and older for HTN once a year free of charge and to implement standardized HTN management measures once the diagnosis is confirmed [18]. The investigators visited households or, through publicity or other channels, asked participants to visit local primary healthcare facilities for the appropriate screening and questionnaires. The current study is based on the 2020 BPHS data in Jiangsu Province to study the prevalence of HTN and the potential dose-response relationships between different anthropometric measures and HTN for individuals aged 65 years and older in this region. Figure 1 shows the specific sampling process and final sample size.

### Socio-demographic and fundamental surveys

During the standardized face-to-face interview, participants were asked to recall whether they had HTN or had taken medication for their BP within the past 2 weeks. Together with possible confounding factors in relevant research, we examined potential associations of HTN with different anthropometric measures through information on participants’ socio-demographic characteristics (age, sex, marital status, and educational level), health behaviors (smoking and drinking behaviors), and medical history [19]. Smoking and drinking behaviors were defined as having smoked or drunk alcohol within 30 days prior to the survey [20]. Thirteen prefecture-level cities in Jiangsu Province were divided into southern Jiangsu (Nanjing, Zhenjiang, Changzhou, Wuxi, and Suzhou), northern Jiangsu (Xuzhou, Lianyungang, Suqian, Yancheng, and Huaian), and middle Jiangsu (Yangzhou, Taizhou, and Nantong) according to the geographical distribution and economic status of each region.

### Data collection and variables

BP and anthropometric measurements were performed in a separate and appropriate room. After 5 minutes of sitting in silence, each participant was required to take 2 BP measurements with standard mercury sphygmomanometers.

According to the U. S. Joint National Committee and Chinese guidelines for the management of HTN (2018) [21,22], HTN is defined as meeting one of the following criteria: SBP ≥ 140 mmHg and/or diastolic blood pressure (DBP) ≥ 90 mmHg, or self-reported use of antihypertensive medication within the past 2 weeks. Other types of HTN (i.e., pre-HTN, isolated high SBP, stage 1 HTN, stage 2 HTN, and stage 3 HTN) were defined with reference to the Chinese guidelines for the prevention and treatment of HTN [22]. When a participant’s SBP and DBP fell into adjacent intervals, the higher category was adopted [22].

For height and weight measurements, individuals were asked to remove their shoes, hats, and jackets to ensure accuracy. WC was measured at minimal breathing. Each trained investigator used standard protocols and techniques to minimize measurement bias. BMI, WtHR, body roundness index (BRI), conicity index (COI), and a body shape index (ABSI) were used in this study and were calculated as follows [13,20,23].

(1) BMI= weight (kg)/height^2^ (m^2^)

(2) WtHR= WC (cm)/height (cm)

(3) BRI= 364.2–365.5[1–π^2^ WC^2^ (m^2^) height^2^ (m^2^)]^1/2^

(4) COI= WC (m)/[weight (kg)/height (m)]^1/2^

(5) ABSI= WC (m)/[BMI^2/3^ (kg/m^2^)/height^1/2^ (m)]

### Statistical analysis

All categorical variables were expressed as percentages, and continuous variables were expressed as mean± standard deviation. Considering the differences in the distribution between the sample and our total population, we standardized the prevalence of HTN by age and sex. The Sixth Census of Chinese Population data was used as the basis for standardization treatment [24]. Markov chain Monte Carlo was used for the interpretation and treatment of missing data on educational level, marital status, and physical work demands [25]. Univariate and multivariate logistic regression analyses were used to estimate the relationships between different anthropometric indices and HTN. We used a restricted cubic spline regression to estimate the non-linear associations among different anthropometric indices and HTN. For balancing the best fit in the spline curve for different anthropometric indices and HTN, a minimum number of knots between 3 and 7 was chosen based on the Akaike information criterion. However, if the different knots were within 2 units of each other, the smallest number of knots was chosen [26]. To further investigate the ability of BMI in combination with other anthropometric measures to assess HTN risk, we established the model with a BMI of 28 kg/m^2^ combined with cut-off points for other anthropometric measures determined by cubic spline regression as the dividing line [27]. Pearson correlation analysis was performed for the anthropometric indices. Data manipulation and statistical analysis were performed using R version 4.1.3 (R Foundation for Statistical Computing, Vienna, Austria). In all analyses, p-values based on two-tailed statistical tests < 0.05 were considered significant.

### Ethics statement

This study was conducted according to the guidelines laid down in the Declaration of Helsinki and all procedures involving research study participants were approved by the Medical Ethics Committee of Jiangsu Provincial Center for Disease Control and Prevention. Informed consent was obtained from all participants included in the study.

## RESULTS

### Basic characteristics of sampled participants

A total of 1,037,511 individuals were recruited for this study. After screening by the criteria shown in Figure 1, a sample size of 944,760 individuals (440,772 male with a mean age of 72.9± 6.0 years and 503,988 female with a mean age of 73.2± 6.3 years) was generated for analysis. The basic characteristics of participants included in the study population are shown in Table 1. Significant differences were found between males and females for all characteristics (p< 0.001). The values or percentages of all basic characteristics of females were significantly higher than those of males, except for weight, height, WC, DBP, WtHR, educational level, and smoking and drinking behaviors (p< 0.001).

### District- specific and sex-specific prevalence of hypertension

**Figure 2.**
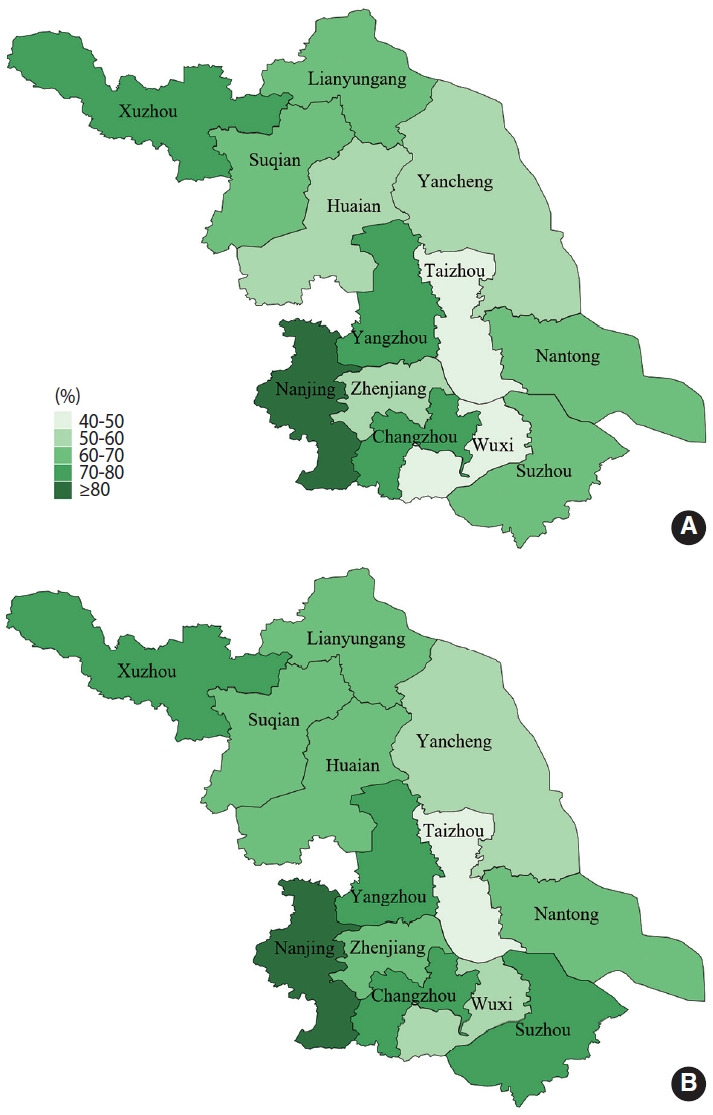
Prevalence for hypertension stratified by sex (A: male, B: female) and region.

The crude prevalence of HTN among elderly individuals in Jiangsu Province was 64.7% (95% CI, 64.6 to 64.8). The prevalence of HTN remained unchanged after standardization of age and sex data from the Sixth Chinese Census in 2010, as shown in Supplementary Material 1. We also mapped the distribution of HTN prevalence across districts by sex, as shown in Figure 2. Among males, 3 district-level cities—Nanjing, Xuzhou, and Yangzhou—ranked in the top 3, with HTN prevalence rates of 80.3%, 75.0%, and 72.3%, respectively, while Wuxi and Taizhou had relatively low prevalence rates of 47.9%, and 48.9%, respectively. Among females, Nanjing, Xuzhou, and Changzhou had the highest prevalence of HTN, with 81.5%, 78.3%, and 74.0% respectively, while Wuxi and Taizhou had a lower prevalence (50.1 and 49.2% respectively). Geographically, the prevalence of HTN in middle Jiangsu was lower than that in southern and northern Jiangsu, as shown in Supplementary Material 2.

### Associations of anthropometric indices with hypertension

**Figure 3.**
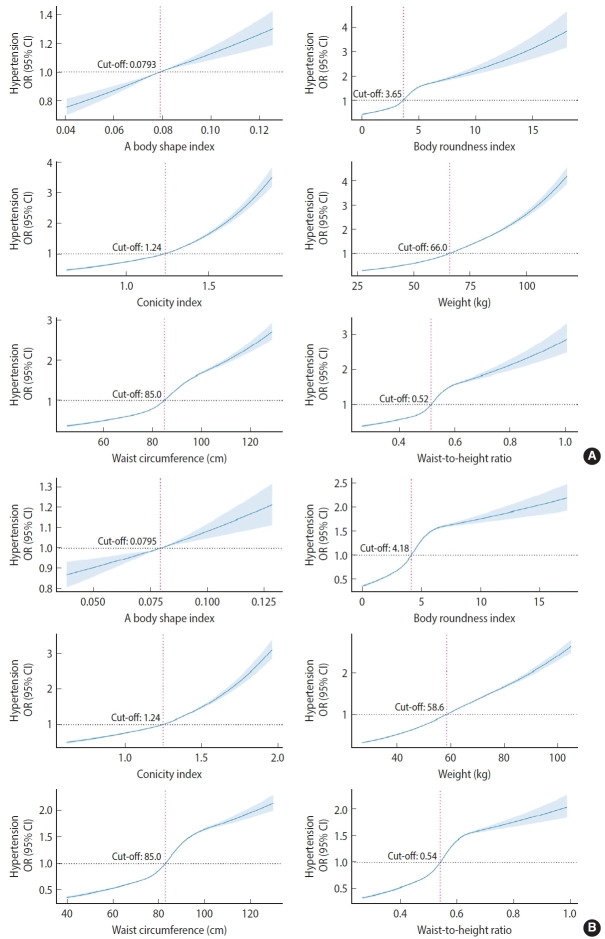
Restricted cubic splines of multiple anthropometric indices associated with hypertension sex (A: male, B: female) - specific elderly populations. OR, odds ratio; CI, confidence interval.

After adjusting for age, physical work demands, educational level, geographic region, marital status, and drinking and smoking behaviors, all anthropometric indices except ABSI (p_nonlinear_=0.3486 in male and p_nonlinear_= 0.7612 in female) showed a significant non-linear positive dose-response association with HTN across sex (p_nonlinear_< 0.001). BMI (cut-off, 24.2 kg/m^2^, not shown in the figure), WC (cut-off, 85.0 cm), and COI (cut-off, 1.24) in both male and female were associated with an increased prevalence of HTN at the same cut-off points. Females had higher cut-off points for positive associations of WtHR and BRI with HTN, while males had a higher cut-off point for a positive association of weight with HTN, as shown in Figure 3.

### Association of the combination of body mass index and other anthropometric indices with hypertension

Correlation analysis of the different anthropometric measures showed that weight had the strongest correlation with BMI (r=0.82, p< 0.001), and COI had the weakest correlation with BMI (r= 0.06, p< 0.001), as shown in Supplementary Material 3. Among participants with BMI < 28 kg/m^2^, abnormal weight (odds ratio [OR], 1.50; 95% CI, 1.49 to 1.52; p< 0.001), WC (OR, 1.63; 95% CI, 1.61 to 1.65; p<0.001), WtHR (OR, 1.62; 95% CI, 1.60 to 1.63; p<0.001), BRI (OR, 1.61; 95% CI, 1.60 to 1.63; p< 0.001), COI (OR, 1.27; 95% CI, 1.26 to 1.28; p< 0.001), or ABSI (OR, 1.11; 95% CI, 1.10 to 1.12; p< 0.001) was positively associated with HTN. The risk of HTN was higher in participants with BMI ≥ 28 kg/m^2^ and other normal or abnormal anthropometric indices, as shown in Table 2.

## DISCUSSION

To our knowledge, this was the largest study ever conducted in Jiangsu Province, China on the prevalence of HTN in individuals aged 65 years and older, with all 13 municipalities serving as surveillance sites. The result showed that 64.7% (95% CI, 64.6 to 64.8) of Jiangsu Province residents aged 65 years and older had HTN. After adjusting for confounders, anthropometric indices such as BMI were significantly associated with the prevalence of HTN. Dose-response relationship analysis showed a non-linear increase in the strength of the association with HTN prevalence as the continuum of anthropometric indices increased in both male and female.

Our research found that the prevalence of HTN among Jiangsu Province residents aged 65 years and older was higher than the results of the 2017 Patient-Centered Event Evaluation of Cardiac Events (PEACE) Million Persons program [28]. However, PEACE was convened among the public through television and newspapers. In contrast, our study used strict sampling, such that more representative results were obtained [29]. The prevalence of HTN among Jiangsu Province’s elderly residents has increased yearly, from 54.4% in 2002 to 64.7% at present [30]. Several factors may have contributed to this phenomenon. First, with the availability of basic public health service programs nationwide and in Jiangsu Province, routine BP measurements have become more accessible to older adults, allowing the identification of those who have not previously been diagnosed with HTN. Second, aging is one of the most important factors contributing to HTN, which was confirmed by the results of this study and other studies [5,31,32]. According to the data of the Seventh Census of Chinese Population in 2020, the percentage of elderly individuals in Jiangsu Province (16.2%) is higher than the national level (13.5%) [15]. Meanwhile, the birth rate in China has declined dramatically. This inverse pyramid demographic structure, combined with the high prevalence of chronic diseases such as HTN, will inevitably lead to a more serious disease burden [33]. HTN has become an increasingly serious public health problem among Jiangsu Province’s elderly residents, and more effective methods should be adopted to prevent and control it. This study also analyzed the geographical distribution of HTN in 13 cities in Jiangsu Province. We found that the regional prevalence of HTN varied widely. The reason for this phenomenon might be the province’s uneven economic development, with significant variation in industrialization and urbanization [34]. It was found that the prevalence of HTN was significantly higher in urban areas in China than in rural ones, which reminded us that developing strategies for the prevention and treatment of chronic diseases such as HTN needs to be integrated with local economic, cultural, and geographic characteristics [28].

Our study found that all 6 anthropometric indices, except ABSI, were positively and non-linearly associated with HTN in both male and female after adjusting for age, physical work demands, educational level, geographic region, marital status, and drinking and smoking behaviors. Previous studies were limited primarily to classifying anthropometric indices such as BMI as categorical variables to be included in the risk model of HTN, thus concealing the trajectory of continuous changes in indices such as BMI and the strength of association with HTN risk [35,36]. This study showed that HTN risk increased significantly when BMI was greater than 24.2 kg/m^2^ in both male and female. Numerous epidemiological studies have found that abnormal BMI is associated with a higher risk of HTN. However, the cut-off points associated with BMI vary from country to country and region to region due to differences in body composition [14,37]. In this study, the BMI cut-off point associated with a higher risk of HTN was 24.2 kg/m^2^, which is consistent with our national target for BMI classification [38]. In addition, we identified cut-off points for other anthropometric indices to provide new scientific support for health screening among the elderly.

Compared with other anthropometric measures, COI and ABSI had relatively low ORs in screening for HTN risk; however, both correlated poorly with BMI. An observational meta-analysis showed that ABSI was significantly less useful than other anthropometric measures in predicting HTN risk [39]. We found a significantly higher risk of HTN when ABSI and COI were combined with BMI. This might be explained by the fact that differences in ABSI and COI do not fully reflect aspects of the metabolic profile, since chronic diseases such as HTN are multifactorial diseases. In addition, both ABSI and COI had low correlations with BMI and could be applied as independent predictors in screening for HTN risk. Therefore, there is a need for subsequent studies to consider the combination of ABSI and COI with BMI when screening older adults for HTN.

This study has several strengths. First, our study was based on a large population with a sample size sufficient to provide statistical reliability. Second, we used restricted cubic splines to reveal nonlinear associations among various anthropometric indices and HTN. However, there were some limitations as well. First, information on the intake of condiments, such as salt, was not collected, which may affect the accuracy of HTN risk factor assessment. Second, we did not obtain information about participants’ awareness, treatment, and control of HTN to provide a systematic and comprehensive picture of HTN among the elderly in general. Finally, this study is a cross-sectional investigation, which lacks strong evidence for causal inference; the findings may be interpreted only as correlations. Relevant cohort studies are in progress to address this issue in the future.

## DATA AVAILABILITY

All data generated or analyzed during this study are included in this article. Further enquiries can be directed to the corresponding author.

## Figures and Tables

**Table 1.** Basic characteristics of participants 65 years and older in Jiangsu Province, China

Characteristics		Male (n=440,772)	Female (n=503,988)	p-value
Age (yr)		72.9±6.0	73.2±6.3	<0.001
Weight (cm)		66.7±11.0	59.7±11.2	<0.001
Height (cm)		164.8±6.6	153.8±6.4	<0.001
WC (cm)		85.6±9.6	84.3±10.2	<0.001
BMI (kg/m^2^)		24.5±3.7	25.2±4.4	<0.001
BRI		3.8±1.2	4.4±1.5	<0.001
COI		1.2±0.1	1.2±0.1	<0.001
WtHR		0.5±0.1	0.5±0.1	<0.001
ABSI		0.1±0.0	0.1±0.0	<0.001
SBP		139.8±18.5	142.1±19.4	<0.001
DBP		83.0±10.2	82.0±10.0	<0.001
Physical work demands				
	Low	51,748 (11.7)	57,895 (11.5)	<0.001
	Middle	223,435 (50.7)	281,787 (55.9)	
	High	74,973 (17.0)	67,246 (13.3)	
	Other	90,616 (20.6)	97,060 (19.3)	
Educational level				
	Primary school or less	201,504 (45.7)	318,499 (63.2)	<0.001
	Junior high school	111,011 (25.2)	62,051 (12.3)	
	High school and higher	29,987 (6.8)	14,693 (2.9)	
	Other	98,270 (22.3)	108,745 (21.6)	
Geographical region				
	Southern Jiangsu	159,749 (36.2)	189,726 (37.6)	<0.001
	Middle Jiangsu	135,108 (30.7)	153,016 (30.4)	
	Northern Jiangsu	145,915 (33.1)	161,246 (32.0)	
Marriage				
	Unmarried	13,720 (3.1)	4,005 (0.8)	<0.001
	Married	391,974 (88.9)	412,916 (81.9)	
	Divorced	30,136 (6.8)	82,037 (16.3)	
	Widowed	1,565 (0.4)	976 (0.2)	
	Other	3,377 (0.8)	4,054 (0.8)	
Drinking behavior^1^		151,731 (34.4)	14,705 (2.9)	<0.001
Smoking behavior^1^		156,690 (35.5)	11,768 (2.3)	<0.001

Values are presented as mean±standard deviation or number (%). WC, waist circumference; BMI, body mass index; BRI, body roundness index; WtHR, waist to height ratio; COI, conicity index; ABSI, a body shape index; SBP, systolic blood pressure; DBP, diastolic blood pressure.

1 Smoking/drinking behavior was defined as having smoked or drunk alcohol within 30 days prior to the survey.

**Table 2.** Associations between hypertension and the combination of BMI with other anthropometric indices^1^

Variables		Non-adjusted	Model I	Model II
BMI (kg/m^2^) & weight (kg)^***^				
	BMI<28 & weight<66.0 (male)/58.6 (female)	1.00 (reference)	1.00 (reference)	1.00 (reference)
	BMI≥28 & weight<66.0 (male)/58.6 (female)	1.94 (1.80, 2.09)	1.77 (1.65, 1.90)	1.77 (1.65, 1.91)
	BMI<28 & weight≥66.0 (male)/58.6 (female)	1.39 (1.38, 1.40)	1.47 (1.46, 1.49)	1.50 (1.49, 1.52)
	BMI≥28 & weight≥66.0 (male)/58.6 (female)	1.70 (1.68, 1.72)	1.77 (1.75, 1.79)	2.07 (2.04, 2.09)
BMI (kg/m^2^) & WC (cm)^***^				
	BMI<28 & WC<85 (male)/83 (female)	1.00 (reference)	1.00 (reference)	1.00 (reference)
	BMI≥28 & WC<85 (male)/83 (female)	1.66 (1.60, 1.72)	1.65 (1.59, 1.71)	1.70 (1.64, 1.77)
	BMI<28 & WC≥85 (male)/83 (female)	1.60 (1.58, 1.61)	1.63 (1.61, 1.64)	1.63 (1.61, 1.65)
	BMI≥28 & WC≥85 (male)/83 (female)	1.85 (1.83, 1.88)	1.89 (1.86, 1.91)	2.19 (2.16, 2.22)
BMI (kg/m^2^) & WtHR^***^				
	BMI<28 & WtHR<0.52 (male)/0.54 (female)	1.00 (reference)	1.00 (reference)	1.00 (reference)
	BMI≥28 & WtHR<0.52 (male)/0.54 (female)	1.56 (1.51, 1.62)	1.58 (1.53, 1.64)	1.67 (1.61, 1.73)
	BMI<28 & WtHR≥0.52 (male)/0.54 (female)	1.64 (1.62, 1.66)	1.62 (1.61, 1.64)	1.62 (1.60, 1.63)
	BMI≥28 & WtHR≥0.52 (male)/0.54 (female)	1.84 (1.81, 1.86)	1.84 (1.82, 1.87)	2.12 (2.09, 2.14)
BMI (kg/m^2^) & BRI^***^				
	BMI<28 & BRI<3.65 (male)/4.18 (female)	1.00 (reference)	1.00 (reference)	1.00 (reference)
	BMI≥28 & BRI<3.65 (male)/4.18 (female)	1.58 (1.52, 1.64)	1.59 (1.54, 1.65)	1.68 (1.62, 1.74)
	BMI<28 & BRI≥3.65 (male)/4.18 (female)	1.63 (1.61, 1.64)	1.62 (1.61, 1.64)	1.61 (1.60, 1.63)
	BMI≥28 & BRI≥3.65 (male)/4.18 (female)	1.84 (1.82, 1.86)	1.85 (1.82, 1.87)	2.12 (2.09, 2.15)
BMI (kg/m^2^) & COI^***^				
	BMI<28 & COI<0.14 (male)/(female)	1.00 (reference)	1.00 (reference)	1.00 (reference)
	BMI≥28 & COI<0.14 (male)/(female)	1.55 (1.52, 1.57)	1.55 (1.52, 1.57)	1.78 (1.75, 1.81)
	BMI<28 & COI≥0.14 (male)/(female)	1.31 (1.30, 1.32)	1.29 (1.28, 1.30)	1.27 (1.26, 1.28)
	BMI≥28 & COI≥0.14 (male)/(female)	1.85 (1.82, 1.88)	1.85 (1.82, 1.88)	2.07 (2.04, 2.11)
BMI (kg/m^2^) & ABSI^***^				
	BMI<28 & ABSI<0.0793 (male)/0.0795 (female)	1.00 (reference)	1.00 (reference)	1.00 (reference)
	BMI≥28 & ABSI<0.0793 (male)/0.0795 (female)	1.51 (1.49, 1.53)	1.51 (1.48, 1.53)	1.74 (1.72, 1.77)
	BMI<28 & ABSI≥0.0793 (male)/0.0795 (female)	1.16 (1.15, 1.17)	1.14 (1.13, 1.15)	1.11 (1.10, 1.12)
	BMI≥28 & ABSI≥0.0793 (male)/0.0795 (female)	1.86 (1.83, 1.90)	1.85 (1.82, 1.89)	2.01 (1.98, 2.05)

Values are presented as odds ratio (95% confidence interval). BMI, body mass index; WC, waist circumference; WtHR, waist to height ratio; BRI, body roundness index; COI, conicity index; ABSI, a body shape index.

1 Model I: adjusted for age and sex; Model II: adjusted for age, sex, physical work demands, education level, geographical region, marriage, drinking behavior, and smoking behavior.

*** p<0.001.

## SUPPLEMENTARY MATERIALS

Supplementary Material 1. Prevalence of different types of hypertensions in the elderly

Supplementary Material 2 Prevalence for hypertension stratified by geographical areas.

Supplementary Material 3 Correlation analysis of anthropometric indices
