# Supplementary material for: Association of multiple anthropometric indices with in 944,760 elderly Chinese people
Source: Epidemiol Health. 2023 Apr 17;45:e2023046. doi: 10.4178/epih.e2023046 (PMC10593587; doi:10.4178/epih.e2023046)
Supplement: Supplementary Material 1. — Prevalence of different types of hypertensions in the elderly [file epih-45-e2023046-Supplementary.docx]

**Supplemental Material**

Supplementary Materia 1. Prevalence of different types of hypertensions in the elderly

| **Characteristics** | **Male (95% CI)** | **Female (95% CI)** | **Total crude (95% CI)** | **Standardized (95% CI)** |
| --- | --- | --- | --- | --- |
| Hypertension | 63.1(63.0-63.2) | 66.1(66.0-66.2) ^***^ | 64.7(64.6-64.8) | 64.7(64.6-64.8) |
| Pre-hypertension | 30.7(30.6-30.8) ^***^ | 28.4(28.3-28.5) | 29.5(29.4-29.5) | 29.5(29.4-29.6) |
| Isolated high SBP | 26.2(26.1-26.3) | 32.8(32.7-33) ^***^ | 29.8(29.7-29.8) | 29.6(29.5-29.8) |
| Stage 1 hypertension | 33.8(33.7-33.9) | 34.3(34.2-34.4) ^***^ | 34.0(34-34.1) | 34.1(34.0-34.2) |
| Stage 2 hypertension | 13.8(13.7-13.9) | 15.8(15.7-15.9) ^***^ | 14.9(14.8-14.9) | 14.8(14.7-14.9) |
| Stage 3 hypertension | 3.9(3.9-4.0) | 5.1(5.0-5.2) ^***^ | 4.6(4.5-4.6) | 4.6(4.5-4.6) |

SBP: systolic blood pressure; 95% CI: 95% confidence interval; ^***^ P value: <0.001


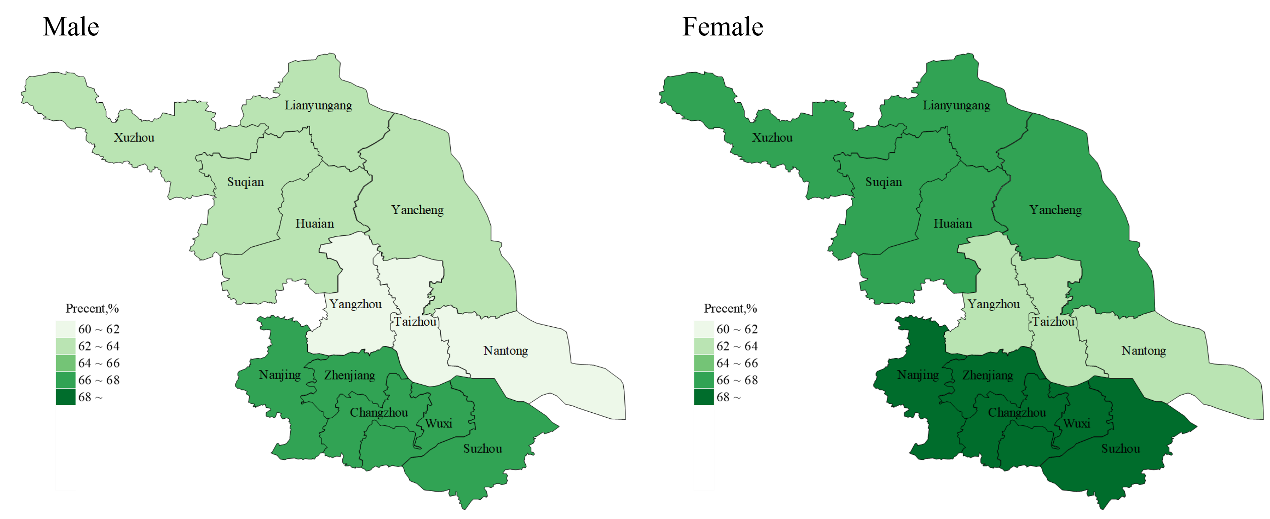


Figure S1

Supplementary Materia 2. Prevalence for hypertension stratified by geographical areas.

Supplementary Materia 3. Correlation analysis of anthropometric indices

|  | BMI | ABSI | BRI | COI | WtHR | Weight | WC |
| --- | --- | --- | --- | --- | --- | --- | --- |
| BMI | 1.000 | -0.264 | 0.732 | 0.063 | 0.728 | 0.816 | 0.712 |
| ABSI | -0.264 | 1.000 | 0.412 | 0.944 | 0.429 | -0.238 | 0.440 |
| BRI | 0.732 | 0.412 | 1.000 | 0.675 | 0.995 | 0.459 | 0.889 |
| COI | 0.063 | 0.944 | 0.675 | 1.000 | 0.692 | 0.032 | 0.701 |
| WtHR | 0.728 | 0.429 | 0.995 | 0.692 | 1.000 | 0.460 | 0.898 |
| weight | 0.816 | -0.238 | 0.459 | 0.032 | 0.460 | 1.000 | 0.705 |
| WC | 0.712 | 0.440 | 0.889 | 0.701 | 0.898 | 0.705 | 1.000 |

WC: waist circumference; BMI: body mass index; BRI: body roundness index; WtHR: waist-to-height ratio; COI: conicity index; ABSI: a body shape index.
